# A Dual Path Model of Work-Related Well-Being in Healthcare and Social Work Settings: The Interweaving Between Trait Emotional Intelligence, End-User Job Demands, Coworkers Related Job Resources, Burnout, and Work Engagement

**DOI:** 10.3389/fpsyg.2021.660035

**Published:** 2021-07-01

**Authors:** Alessio Tesi

**Affiliations:** Department of Information Engineering, University of Pisa, Pisa, Italy

**Keywords:** trait emotional intelligence, work engagement, burnout, healthcare, well-being, path model, social work

## Abstract

Framing the job demands-resources (JD-R) model, the present study deepened how trait emotional intelligence (TEI, i.e., perception about one's own emotional realm) contributes to the work-related well-being of healthcare professionals. A total of 302 healthcare professionals were involved in the study and completed an anonymous self-report questionnaire. The results of the structural equation modeling revealed that TEI was directly and indirectly—mediated by end-user job demands—negatively associated with burnout, and directly and indirectly—mediated by coworkers related job resources— associated with work engagement. According to the health impairment and motivational processes of JD-R, the present study highlights that TEI could targets burnout and work engagement through different paths. The first path revealed that TEI would reduce burnout protecting by the insurgence harmful relationships with service end-users and the second showed that TEI would support work engagement sustaining the development of positive relationship with coworkers.

## Introduction

In the realm of organizational settings, work-related well-being has been conceptualized as both the absence of detrimental condition (e.g., stress, burnout; e.g., Aiello et al., 2012; Usman et al., 2020), and the presence of promotional factors (e.g., work engagement; job satisfaction; e.g., Schaufeli et al., 2002; Anser et al., 2020) which can have a role in fostering employee's work-related health (see Bakker, 2015). The present study is focused on work-related well-being of healthcare workers. Healthcare work can be labeled as “emotional labor” (de Jonge et al., 2008; Hülsheger and Schewe, 2011; Ingram, 2013) as healthcare workers, (i) face emotional demands due to emotionally charged interactions with service recipients (i.e., job-focused emotional labor) and, (ii) should denote a great ability to menage emotions (i.e., employee-focused emotional labor). Since “emotional issues” are particularly salient among healthcare workers, the present study aimed to contribute to the literature that attempt to explain how Trait Emotional Intelligence (TEI; Petrides and Furnham, 2000)—a constellation of emotion related self-perceptions belonging to the personality domain—is related to the well-being of these professionals. According to a series of researches (e.g., Durán et al., 2004; Akhtar et al., 2015; Fiorilli et al., 2019) the present study attempted to confirm that among healthcare professionals the awareness and the ability to manage emotions (i.e., high levels of TEI) can help to lower the emotional breakdown of burnout and support work engagement (WE). Furthermore, little is known on the mechanism *through* which TEI is related to burnout and WE. Framing the job demands-resources (JD-R) model (Bakker and Demerouti, 2017), the present study attempted to address this research gap studying the contribution of two novel specific mediators. In particular, it was hypothesized that high levels of TEI can indirectly, (i) lessen burnout through lessening the disruptiveness of harmful relationships between healthcare professionals and end-users of healthcare service (i.e., end-users job demands; Aiello and Tesi, 2017b); (ii) increase WE through improving positive social interactions with colleagues (i.e., coworkers-related job resources; Aiello et al., 2012). Since the evidence on the role of TEI in healthcare professionals are fragmented through various studies which separately analyzed the “facets” of work-related well-being (e.g., burnout *or* WE), the present study is one of the first attempt in integrating the two perspectives into a comprehensive conceptual dual-path model of work-related well-being of healthcare (Mehta, 2020).

## Hypotheses Development

### “Two Faces of the Same Work-Related Well-Being”: Burnout and Work Engagement

Burnout is defined as a job-related psychological syndrome of emotional exhaustion, depersonalization, and reduced personal accomplishment (Maslach, 1993). Exhaustion refers to depleting one's own emotional resources as a consequence of prolongated stress exposure; depersonalization is an attitude of viewing others—especially users requiring care in human services—with indifference, a lack of closeness and cynicism; reduced personal accomplishment is the lack of a sense of achievement of competence and efficacy regarding the workflow. Despite burnout originally being composed of these three factors, further research (e.g., Lee and Ashforth, 1996; Demerouti et al., 2010; Taris et al., 2017) pointed out exhaustion and depersonalization as the “core” dimensions of burnout. Indeed, within the healthcare profession, these two dimensions especially capture the consequences of detrimental social relationships experienced by professionals and service recipients as burnout “had its roots in care-giving and service occupations, in which the core of the job was the relationship between provider and recipient” (Maslach et al., 2001, p.400). WE has been defined as “a positive and fulfilling work-related state characterized by vigor, dedication, and absorption” (Schaufeli et al., 2002, p.74). Vigor, the energetic component of WE, refers to the level of energy and resilience of workers. Absorption is the affective component of WE and concerns the level of enthusiasm, significance, pride, challenge, and inspiration that workers can feel—it is referred to as the state of being fully concentrated and deeply absorbed in work tasks.

Under the framework of the JD-R model (Bakker and Demerouti, 2007, 2017), work-related well-being could be conceived as a multifaced, desirable status comprising of the absence of health-detrimental indicators, such as burnout, and the presence of health-inducing status such as WE (Bakker et al., 2014; Schaufeli, 2017; Mehta, 2020). Thus, burnout and WE can be considered “*two faces of the same coin”* of work-related well-being. Burnout and WE are negatively correlated (Taris et al., 2017), both constructs overlap only partially, conceptually and empirically (Demerouti et al., 2010). According to this notion and the research based on the JD-R model (e.g., Schaufeli et al., 2002; Hakanen et al., 2006; Bakker et al., 2014) burnout could be considered the “negative pole” of work-related well-being, while WE could be considered “the positive pole.” The present study aims to integrate both constructs as two differentiated dependent variables of a model that attempt to deepen the factors that contribute to the well-being at work of healthcare professionals.

### The Role of Trait Emotional Intelligence

TEI, also called trait emotional self-efficacy (Petrides and Furnham, 2000), was defined as “a constellation of emotional self-perception” (Petrides, 2011, p.656). It is located in the personality domain and represents one's perception of their own ability to manage emotions (Petrides et al., 2016). TEI is composed of four dimensions—emotionality, self-control, sociability, and well-being—that are self-perceptions that enable emotional capability, willpower, sociability, and adaptability, respectively (Petrides, 2009). Several studies linked TEI with work-related well-being (Petrides and Furnham, 2006; Schutte and Loi, 2014; Di Fabio and Kenny, 2016). Scholars highlighted that emotional intelligence could be a key factor for sustaining the well-being of helping professionals (Ingram, 2013). As found in previous studies, since burnout is mainly characterized by emotional exhaustion, a greater ability to manage one's own negative emotions (i.e., TEI), could prevent the rising of burnout among healthcare workers (e.g., Durán et al., 2004; Aiello and Tesi, 2017a; Fiorilli et al., 2019). Furthermore, Akhtar et al. (2015) found that TEI was also directly, positively associated with WE in a large sample of workers, over and beyond other personality factors. Workers with high levels of TEI are more prone to apply greater energy and effort at work, because they can better manage their emotions and social interactions.

*Hypothesis 1: TEI was directly negatively associated with burnout (a) and positively associated with WE (b)*.

### The Mediational Role of End-User Job Demands and Coworkers-Related Job Resources

Under the framework of JD-R model (Bakker and Demerouti, 2007, 2017), job demands are those physical, psychological, social, and organizational aspects of the job that present costs to workers, potentially impairing their health. Different classes of job demands have been identified, like those related to organizational management or policies (e.g., role conflicts, role ambiguity, work-family conflicts). Job demands fuel an health-impairment process that gradually leads to experiencing stress and burnout (Bakker and Demerouti, 2017). Since the healthcare professions are considered “high touch emotional labors” (Hülsheger and Schewe, 2011), the present study focused in examining the role of emotional job demands (de Jonge et al., 2008). Emotional job demands can refer to, (i) the perceived emotional charge of the job and, (ii) be related to the development of physical and emotionally harmful relationships between the healthcare professionals and service end-users (Aiello and Tesi, 2017a). Specifically, the present study aimed at examining how much TEI could contribute to healthcare workers' well-being through empowering the quality of the relations. Thus, it was hypothesized a particular contribution of the second type of emotional job demands. These types of job demands were labeled here as “end-user job demands”. End-user job demands specifically intercept the detrimental nature of the relationships between healthcare workers and service end-users. These harmful relationships are often characterized by unceasing requests and sometimes by physical and psychological violence (Littlechild, 2005; Padyab et al., 2013). High levels of TEI could help in facing the emotionally harmful interactions with end-users. Moreover, the JD-R model's health impairment process suggests that emotional job demands were related with the development of burnout (de Jonge et al., 2008). Although it has not been tested, literature suggests an interweaving between TEI, end-users job demands, and burnout. Trying to address this research gap, it was hypotesized that TEI was associated with end-user job demands that, in turn, were associated with burnout.

*Hypothesis 2: TEI was indirectly, negatively associated with burnout mediated by end-user job demands*.

**Figure 1:**
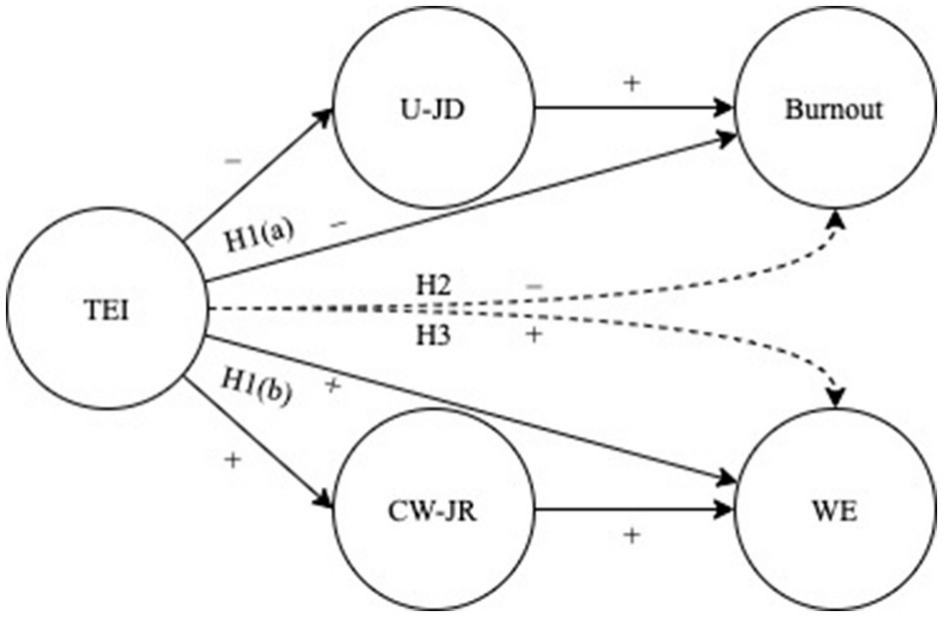
Graphical summary of study's conceptual model and hypotheses. (+) and (−) signs indicate the positive and negative hypothesized associations, respectively. H1(a) = hypothesis 1(a); H1(b) = hypothesis 1(b); H2 = hypothesis 2; H3 = hypothesis 3. TEI, trait emotional intelligence; U-JD, end-user job demands; CW-JR, coworkers related job resources; WE, work engagement. Continuous lines = direct relationship; discontinuous lines = indirect relationship.

Job resources are those physical, psychological, social, and organizational aspects of the job that stimulate personal growth and sustain the achieving of work objectives (Bakker and Demerouti, 2007, 2017). Different classes of job resources include job control, performance feedback and skill variety. The JD-R model posited that the motivational process (i.e., independent from the health impairment process; Bakker and Demerouti, 2017) provides that job resources fuel WE, workflow, and enjoyment at work (Bakker et al., 2014; Bakker and Demerouti, 2017), even among healthcare professionals (García-Sierra et al., 2016; Aiello and Tesi, 2017b). The present study is focused on job resources related to the development of positive relations with coworkers that have been called “coworkers-related job resources.” These resources can be identified as those implying positive, functional, and supportive contacts with colleagues and supervisors (e.g., social support, group cohesion and quality of communication processes with coworkers; Aiello et al., 2012). Coworkers-related job resources play a significant role in promoting the work-related well-being of healthcare professionals (e.g., Giannetti and Tesi, 2016; Pekaar et al., 2018). Studies found that coworkers' social support was associated with WE and work performance in nurses (García-Sierra et al., 2016), particularly when they also have high levels of TEI (Toyama and Mauno, 2017). Thus, the reported literature suggest the possible existence of a resourceful path that links emotional competence (i.e.,TEI) with the development of positive relations with coworkers that, in turn, can be promotional of WE. For a summary of the study's hypotheses see Figure 1.

*Hypothesis 3: TEI was indirectly, positively associated with WE mediated by coworkers-related job resources*.

## Method

### Participants and Procedure

Ten Italian healthcare organizations (social cooperatives) were approached, potentially involving a total of 419 healthcare professionals. In accordance with the managers of these organizations, an email was sent to each potential participant asking them to voluntarily take part in a research on “well-being of healthcare professionals.” Each participant was also allowed to involve colleagues to take part in the research (snowball sampling). A total of 302 professionals answered and accepted to participate in the study (response rate: 72.10%). The sample was composed of 243 women and 59 men. The age distribution of 55 participants ranged from 18 to 30 years, 105 from 31 to 40 years, 85 from 41 to 50 years, 52 from 51 to 60 years, and 5 from 61 to 70 years. Twenty-one participants had completed primary school, 81 completed high school, 111 had bachelor's degree, and 81 had master's degree. As for work seniority, 88 participants ranged from 1 to 5 years of seniority, 61 ranged from 6 to 10 years, 95 from 11 to 20 years, 47 from to 21 to 30 years, 11 from 31 to 45 years. Those involved in different healthcare professions included 116 social workers, 105 healthcare assistants, 54 social educators, 24 nurses, and three psychologists. Participants gave their informed consent and completed an anonymous self-reported online questionnaire in several scheduled sessions, comprised of a maximum of 30 participants per session. To reduce possible response-bias, data collection was supervised by the researcher; in each session it was clearly stated to the participants that their answers were strictly anonymous, and was treated exclusively for research purposes. For this reason, participants were also recommended to answer to each question spontaneously. This study is a part of comprehensive research on “well-being in the Italian helping professions” (e.g., Giannetti and Tesi, 2016; Aiello and Tesi, 2017a,b) and it was conducted in line with the Italian Association of Psychology (AIP) ethical standards.

### Measures

#### Trait Emotional Intelligence

TEI was measured using the Italian version (Di Fabio and Palazzeschi, 2011) of the Trait Emotional Intelligence Questionnaire-Short Form (Cooper and Petrides, 2010). The scale was composed of a total of 26 items with a 7-point Likert response format (from 1 = completely disagree, to 7 = completely agree) for measuring different dimensions comprising TEI: emotionality (six item; i.e., “Expressing my emotions with words is not a problem for me”; α = 0.60), self-control (six item; i.e., “I'm usually able to find ways to control my emotions when I want to”; α = 0.60), sociability (four item; i.e., “I can deal effectively with people”; α = 0.54), and well-being (six item; i.e., “On the whole, I'm pleased with my life”; α = 0.72).

#### End-User Job Demands

For measuring end-user job demands, the subscale dealing with users' complaints of the Emotional Job Demands Scale was used (Xanthopoulou et al., 2013; Aiello and Tesi, 2017a). The scale was composed of three items with a 5-point Likert response whose format ranged from “1 = never,” to “5 = always.” The items were the following: “In your work do you deal with clients who incessantly complain, although you always do everything to help them?” “In your work, do you have to deal with demanding clients?” “Do you have to deal with clients who do not treat you with the appropriate politeness and respect?” The scale alpha was of 0.75.

#### Coworkers-Related Job Resources

Coworkers-related job resources were measured using the Evaluation of Psychosocial Risks Questionnaire (Aiello et al., 2012). In particular, were used three specific subscales for intercepting various facets of coworkers' support, coworkers' social support (three items; i.e., “When I have work issues colleagues are well-disposed to listen to me,” α = 0.84), group cohesion (three items, i.e., “In my workgroup we help each other,” α = 0.80), and quality of communication processes with coworkers (four items, i.e., “Even in troublesome working situations we are able to maintain a good communication,” α = 0.88). Participants were asked to answer using a 6-point Likert scale ranging from “1 = completely disagree,” to “6 = completely agree.”

#### Burnout

Burnout was measured with the Italian version (Sirigatti and Stefanile, 1993) of the Maslach Burnout Inventory (Maslach et al., 1996). The measure of the “core” of burnout was composed of emotional exhaustion (nine items; i.e., “I feel emotionally exhausted in my work,” α = 0.87), and depersonalization (five items; i.e., “I do not really care what happens to my assisted users,” α = 0.69). Participants were asked to answer using a 6-point Likert scale ranging from “0 = never,” to “6 = always.”

#### Work Engagement

We was measured using the Italian version (Balducci et al., 2010) of the Utrecht Work Engagement Scale (Schaufeli et al., 2006). WE is assessed measuring the following three dimensions: vigor (three items, i.e., “At my work, I am bursting with energy,” α = 0.85), dedication (three items, i.e., “I am enthusiastic about my job,” α = 0.89), and absorption (three items, i.e., “I am immersed in my job,” α = 0.75). Participants used a 7-point Likert scale ranging from “0 = never,” to “6 = always.”

### Data Analysis Procedure

The hypotheses were tested with SPSS AMOS 21 running a structural equation model (SEM) with a maximum likelihood (ML) estimation. SEM approaches are preferred to a classical regression approach because it permits testing complex path models, acknowledging and verifying measurement errors. In the current study was tested a model with five latent variables, TEI (i.e., observed variables: emotionality, self-control, sociability, and well-being), end-user job demands (i.e., observed variables: the 3 items of DUC scale), coworkers-related job resources (i.e., observed variables: coworkers' social support, group cohesion, and quality of commutation processes), burnout (i.e., observed variables: emotional exhaustion and depersonalization), and WE (i.e., observed variables: vigor, dedication and absorption), respectively. The goodness of the model fit was evaluated with the following fit indexes: the χ^2^, the χ^2^/*df* , the comparative fit index (CFI), the root mean square error of approximation (RMSEA), and standardized root mean square residual (SRMR). A model is considered to have a reasonable fit to the data if χ^2^*/df* is less than three, a CFI above 0.90 is considered acceptable, RMSEA and SRMR below the cut-off of 0.08 are considered acceptable (Hooper et al., 2008). The study's hypothesss 1 (a,b) provided the testing of the direct association between TEI and burnout, and between TEI and WE. Hypotheses 2 and 3 provided the testing of the indirect effect of TEI (i.e., predictor) on burnout (i.e., outcome) mediated by end-user job demands (i.e., mediator), and the indirect effect of TEI (i.e., predictor) on WE (i.e., outcome) mediated by coworkers-related job resources (i.e., mediator). To conclude that an indirect effect occurred, one must establish the existence of a direct path between the predictor and the mediator, and a path of association between the mediator and the outcome, acknowledging in the same model a direct path between the predictor and outcome (Holmbeck, 1997; MacKinnon, 2008).

## Results

### Preliminary Analysis

Descriptive statistics, skewness, kurtosis, and correlational analysis were performed using SPSS 27 on measured variables and reported in Table 1. Since all observed variables ranged from +/– 2 on skewness and kurtosis, data normal distribution was assumed. A preliminary exploration of the issue of common method bias was done with Harman's single-factor test (Podsakoff et al., 2003). A principal component analysis with a non-rotated solution was performed inserting all the study's measured variables. Results highlighted that the first component extracted explained 37.97% of total variance; being beyond the established threshold (50%; Rodríguez-Ardura and Meseguer-Artola, 2020) no common method bias issue was identified.

**Table 1:** Means, standard deviations, skewness, kurtosis, and correlations among variables.

**Variable**	**M**	**SD**	**Skewness**	**Kurtosis**	**1**	**2**	**3**	**4**	**5**	**6**	**7**	**8**	**9**	**10**	**11**	**12**
1. Emotionality	5.58	0.79	−0.67	0.54												
2. Self-control	4.99	0.89	−0.03	−0.37	0.50											
3. Sociability	4.89	0.85	−0.22	0.06	0.54	0.50										
4. Well-being	5.72	0.89	−0.63	−0.16	0.51	0.57	0.51									
5. End-user job demands	3.37	0.88	−0.01	−0.60	−0.17	−0.17	−13	−0.17								
6. Co-workers social support	3.21	1.57	0.00	−0.98	0.22	0.27	0.25	0.27	−0.05^†^							
7. Group cohesion	3.20	1.48	−0.08	−0.78	0.22	0.26	0.27	0.24	−0.08^†^	0.83						
8. Quality of communication processess	3.08	1.54	−0.12	−0.87	0.20	0.33	0.25	0.31	−0.05^†^	0.69	0.74					
9. Emotional exhaustion	2.11	1.34	0.72	0.01	−0.37	−0.42	−0.32	−0.37	0.38	−0.26	−0.27	−0.36				
10. Depersonalization	1.21	1.13	1.16	1.20	−0.45	−0.37	−0.31	−0.32	0.34	−0.14^*^	−0.15^*^	−0.24	0.66			
11. Vigor	4.31	1.21	−0.86	0.75	0.29	0.41	0.29	0.43	−0.20	0.35	0.34	0.43	−0.53	−0.37		
12. Dedication	4.62	1.30	−1.23	1.51	0.32	0.35	0.34	0.43	−0.20	0.35	0.35	0.43	−0.47	−0.42	0.83	
13. Absorption	4.33	1.18	−0.74	0.58	0.30	0.23	0.24	0.25	−0.17	0.27	0.23	0.24	−0.29	−0.27	0.63	0.66

*All correlations are significant at p < 0.001, excluding*

“^*^”significant at p < 0.05 and

*“^†^”no significant*.

### Model Testing

**Figure 2:**
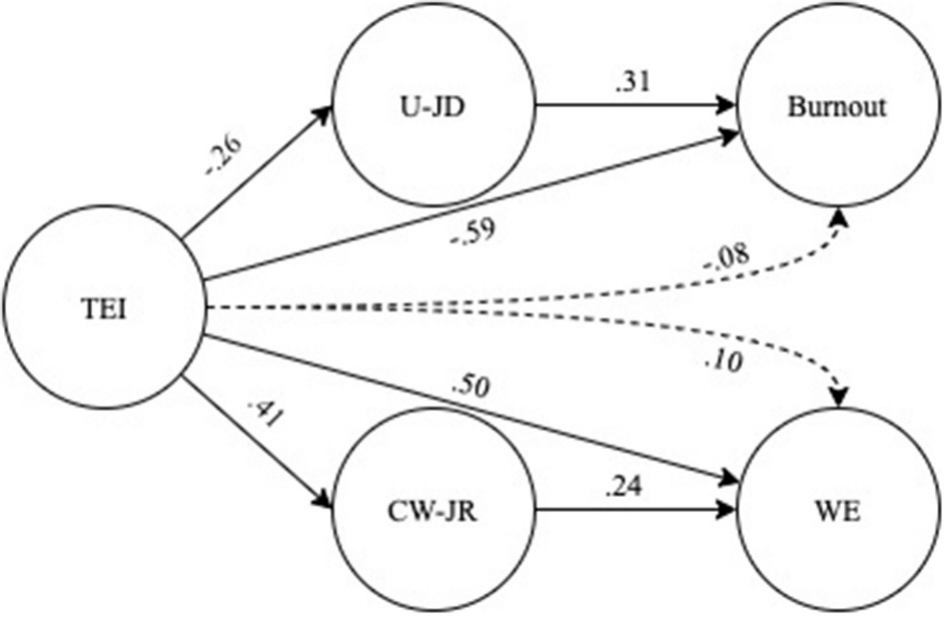
Summary of the results of structural equation model. TEI, trait emotional intelligence; U-JD, end-user job demands; DUC, dealing with users' complaints; CW-JR, coworkers related job resources; WE, work engagement. Continuous lines = direct relationship; discontinuous lines = indirect relationship. All direct and indirect paths coefficients were statistically significant at *p* < 0.001.

The tested model showed a satisfactory fit to the data (χ^2^ = 216.60, *df* = 84, χ^2^/*df* = 2.58, CFI = 0.94; RMSEA = 0.07 (90% CI: 0.06, 0.08); SRMR = 0.06). Factor loadings of the observed variables ranged from a minimum of 0.68 to a maximum of 0.93. Hypotheses 1(a) and 1(b) were confirmed. A negative and direct association between TEI and burnout (*b* = −.59, *p* < .001), and a positive association between TEI and WE (*b* = 0.50, *p* < .001) were found. Hypotheses 2 and 3 were confirmed. A negative association between TEI and end-user job demands (*b* = −0.26, *p* < 0.001), and a positive association between end-user job demands and burnout (*b* = .31, *p* < .001) were found. Moreover, we found a positive association between TEI and coworkers-related job resources (*b* = 0.41, *p* < 0.001), and between coworkers-related job resources and WE (*b* = 0.24, *p* < 0.001). The hypothesized indirect effects of TEI on burnout and WE were tested generating a 95% bootstrap bias-corrected percentile confidence interval with 5000 bootstrap samples (Cheung and Lau, 2008). The analysis revealed that the hypothesized negative indirect effect of TEI on burnout mediated by end-user job demands was significant (*b* = −0.08, *p* < 0.001, 95% CI: −0.14, −0.04), and that also the hypothesized positive indirect effect of TEI on WE mediated by coworkers-related job resources was significant (*b* = 0.10, *p* < 0.001, 95% CI: 05, 0.16). The model^1^ (Figure 2) explained a 53% (*R*^2^ = 0.53) of burnout and a 40% (*R*^2^ = 0.40) of WE total variance.

## Discussion

As hypothesized (1a, 1b) and in line with previous studies (e.g., Baik and Yom, 2012; Toyama and Mauno, 2017; Fiorilli et al., 2019), the results revealed that TEI was directly and negatively associated with burnout and positively associated with WE. TEI concerns people's capacity to manage emotions within social interactions (Petrides, 2009; Petrides et al., 2016). From literature it is well-known that healthcare professionals, as “emotional-labor” (Brotheridge and Grandey, 2002), are at a high risk of burnout and that higher levels of TEI can protect these professionals from this disruptive syndrome. In particular, higher levels of TEI could help to cope with the emotionally exhausting feelings of burnout (Durán et al., 2004; Fiorilli et al., 2019). Not only could TEI lessen burnout, but also could have a role in fostering WE of these professionals. Workers can apply greater energy and efforts at work because they feel they are emotionally capable to cope with job issues (Akhtar et al., 2015).

Since little is known on the processes that link TEI with burnout and WE, the present study filled this research gap, revealing a dual path model in which was deepened the role of end-users job demands and coworkers-related job resources as mediators. In line with the hypothesis 2, and according to the health impairment process of the JD-R model, the first path revealed that TEI would indirectly reduce burnout by lessening the health-impairment effect of job demands derived from the excessively demanding relationship with service end-users (i.e., end user job demands). High levels of TEI meant proper self-managing of emotional and social issues; this could prevent the development of harsh relationships with service users and thus prevent burnout (e.g., Maslach et al., 2001; Brotheridge and Grandey, 2002; Littlechild, 2005; Padyab et al., 2013; Montgomery et al., 2015). The results also confirmed hypothesis 3, finding that TEI could sustain proficient and empathetic relationships with coworkers (e.g., coworkers-related job resources) that, in turn, can improve WE. This result is in line with other studies (García-Sierra et al., 2016; Toyama and Mauno, 2017) and with the motivational process of the JD-R model. As far as the study's model is concerned, the results revealed that TEI acts as a promotional factor that can indirectly improve healthcare professionals' WE through ameliorating their relationships with coworkers. On the whole, the results of the current study (Figure 1) support the notion that in healthcare settings TEI could target two independent paths of work-related well-being. The first path suggests that TEI could reduce burnout by lessening the development of harmful relationships with service end-users, and the second suggests that TEI could support WE by ameliorating the relationships with coworkers.

As far as the study's limitations, one's should outline that the directionality of the association's paths should be interpreted with caution since the present study was planned with a cross-sectional design. Future longitudinal studies can address this limitation. The study's sample was unbalanced in terms of gender, although this is typical in many healthcare and social work settings (e.g., Durán et al., 2004; Aiello and Tesi, 2017b; Toyama and Mauno, 2017). The current study considers two classes of job demands and resources (e.g., end-user job demands and coworkers-related job resources) particularly salient for the well-being of the healthcare professionals (de Jonge et al., 2008; García-Sierra et al., 2016) and susceptible to be influenced according to TEI's levels. However, other kinds of job demands/resources, as well as personal resources could be considered in future studies for deepening the complexity of the study's model. Future studies, according to the JD-R model, can consider the possible joint effects of the various job demands and job resources on healthcare professionals' well-being.

### Practical Implication

From the present study arises that emotional intelligence is a key individual difference in emotional labor, characterizing social work and healthcare professions (Ingram, 2013; Pekaar et al., 2018). In particular, the assessment of emotional intelligence could be implemented as part of the personnel selection process by the management of healthcare organizations. Since the current study highlights that healthcare professionals' work-related well-being (i.e., in terms of low burnout and high WE) is also a matter of how they can manage emotions and relations with service recipients and coworkers, intervention for promoting the TEI could be implemented. Sustainable group interventions (Campo et al., 2015) can be tailored for being applied as a part of training programs dedicated to fostering healthcare professionals' skills (e.g., Grant et al., 2014; Kozlowski et al., 2018; Meng and Qi, 2018). These interventions can increase the awareness about self-reported emotional intelligence, the knowledge, regulation, expression of emotions, and thus promote better relationships with end-users and co-workers. TEI intervention can also support health promotion and gains in other life domains; TEI could reduce psychological disorders (i.e., depression), negative health-related behavior (e.g., smoking, alcohol consumption), and promote positive one's (e.g., regular exercising, healthy diet; Campo et al., 2015).

## Data Availability Statement

The dataset of this study is not publicly available following local legal and privacy restrictions (Italian Data Protection Code; Legislative Decree No. 196/2003).

## Ethics Statement

The author states that the procedures of this study are in line with the Italian Association of Psychology (AIP) ethical standards. The participants provided their written informed consent to participate in this study.

## Author Contributions

The author confirms being the sole contributor of this work and has approved it for publication. AT designed the study, did data collection, statistical analyses, wrote, and revised the manuscript.

## Conflict of Interest

The author declares that the research was conducted in the absence of any commercial or financial relationships that could be construed as a potential conflict of interest.

## Footnotes

^1^To further verify the goodness of the final model (FM, Figure 2), FM was compared with an alternative model (AM) in which, starting from FM, were also drawn two new paths, i) between end-users job demands and WE, and, ii) between coworkers-related job resources and burnout. In line with the expectations, (i.e., the independence between the health impairment process and the motivational process of JD-R model; Bakker and Demerouti, 2017), these two paths were not statistically significant. Model fit of AM was of, χ^2^ = 211.65, *df* = 82, χ^2^*/df* = 2.58, CFI = 0.94; RMSEA = 0.07 (90% CI: 0.06, 0.08); SRMR = 0.05. Since the chi square differences test between AM and FM was not significant Δχ^2^(2) = 4.94, *p* = 0.09, the parsimonious model (FM) was preferred.
